# Environmental Exposures Influence Fetal Brain Growth and Risk of Neonatal Brain Injury in Congenital Heart Disease

**DOI:** 10.21203/rs.3.rs-8206641/v1

**Published:** 2025-12-03

**Authors:** Lesje DeRose, Megan Martin, Elizabeth George, Karla Luna Silva, Flora Nuñez-Gallegos, Martina Steurer, Duan Xu, Patrick McQuillen, Shabnam Peyvandi

**Affiliations:** University of California San Francisco Benioff Children’s Hospitals; Stanford University; University of California San Francisco; University of Pennsylvania; University of California San Francisco Benioff Children’s Hospitals; University of California San Francisco Benioff Children’s Hospitals; University of California San Francisco; University of California San Francisco Benioff Children’s Hospitals; University of California San Francisco Benioff Children’s Hospitals

**Keywords:** congenital heart disease, neurodevelopment, brain growth, environmental exposures, social determinants of health

## Abstract

**Background:**

Neurodevelopmental impairments are common in congenital heart disease (CHD) and fetal brain volume is an important predictor of outcomes. Social determinants of health (SDOH) and environmental factors influence brain growth in other populations and likely play a neurodevelopmental role in CHD. This study evaluated the influence of SDOH and environmental factors on fetal and neonatal brain volume, growth, and risk of brain injury in CHD.

**Methods:**

This prospective single-center longitudinal cohort study enrolled fetuses with severe CHD to undergo third-trimester fetal and preoperative brain MRIs. Controls underwent third-trimester brain MRIs. Participants completed SDOH and environmental exposure surveys. Fetal and neonatal brain volumes, brain growth, and presence of white matter injury (WMI) were assessed.

**Results:**

57 CHD patients and 24 controls were enrolled, resulting in 33 fetal and 44 neonatal MRIs in the CHD group and 21 fetal control MRIs. Several SDOH and environmental factors, including maternal smoking, were associated with smaller brain volume and slower brain growth in CHD but not in controls. With CHD, repeated-measures analysis showed smaller fetal brain volume (coeff: −13.3, 95%CI: −25.5,−1.1 p = 0.03) and slower growth (coeff: −2.5, 95%CI: −5.0, −0.07, p = 0.04) with exposure to any risk factor. CHD subjects from high Childhood Opportunity Index neighborhoods had lower odds of moderate to severe preoperative WMI (OR = 0.16, 95%CI: 0.03, 0.9, p = 0.04).

**Conclusions:**

SDOH and environmental exposures influence fetal brain growth and preoperative brain injury risk in CHD. These results highlight additive environmental prenatal risks which may be amenable to early intervention.

## Introduction

Neurodevelopmental (ND) impairments are a significant comorbidity in patients with congenital heart disease (CHD). Studies have shown that postnatal risk factors only account for one-third of the variance in ND outcomes [1]. Moreover, infants with CHD have differential brain development beginning in fetal life [2] and are at increased risk for acquired brain injury in the neonatal period [3–5]. CHD patients demonstrate smaller total and regional brain volumes, impaired neuroaxonal development and metabolism, and altered brain growth trajectory beginning in utero as well as higher rates of acquired brain injury in the form of white matter injury (WMI) after birth [6–10]. Importantly, these early neuroimaging markers in the fetal and neonatal period have been independently linked to ND outcomes in CHD patients and appear to have a greater impact on outcomes compared to postnatal factors, making them important neuroimaging markers to track [11–13]. Thus, the fetal and neonatal time periods deserve further investigation to fully understand risk factors for adverse neurodevelopmental outcomes, which may identify potential neuroprotective interventions.

Brain dysmaturation in patients with CHD is thought to be multifactorial in etiology, with genetic, cardiovascular, and fetal environmental components [14–16]. Some adverse fetal environmental factors, such as maternal stress, have been linked with fetal brain growth and development in CHD populations [17]. In other conditions such as preterm birth, factors related to social determinants of health (SDOH) and other exposures in the fetal environment have been associated with fetal total brain volume (TBV) and ND [18–21]. SDOH have been associated with a wide range of ND outcomes in the general population as well as in the CHD population [22–25]. With early intervention, ND discrepancies related to SDOH factors may be modifiable as recently demonstrated by anti-poverty intervention initiatives [26].

The primary aim of this study was to determine if SDOH and fetal environmental exposures influence fetal brain growth and risk of postnatal pre-operative brain injury in severe CHD. We hypothesized that factors related to a lower socioeconomic status and exposure to adverse environmental factors would be associated with slower fetal brain growth and higher risk of WMI.

## Methods

This data was collected as part of a prospective longitudinal cohort study enrolling pregnant individuals with a prenatal diagnosis of severe CHD expected to undergo a neonatal cardiac operation at the University of California San Francisco (UCSF) between 2017–2022. Participants were enrolled to undergo a fetal brain MRI at late gestation followed by postnatal pre-operative brain MRI after birth. In addition, health pregnancy individual with no fetal anomalies and normal fetal ultrasound and echocardiograms were enrolled from the low-risk obstetrical clinic at UCSF between 2017–2019 as a control group [27, 28]. Those with a fetal diagnosis of genetic or extracardiac abnormalities, twin gestation, growth restriction, or significant uteroplacental disease such as preeclampsia and maternal disease (diabetes and hypertension requiring medication use) were excluded. The study protocol was approved by the institutional review board on human research at UCSF and informed consent was obtained from all participants. The CHD cohort predominantly included fetuses with either d-transposition of the great arteries (TGA) or single ventricle physiology (SVP). TGA was defined as great vessel malposition with the aorta arising from the right ventricle and the pulmonary artery arising from the left ventricle with or without a ventricular septal defect. SVP was defined as the absence of one of two functioning ventricles requiring a palliative surgical intervention for survival in the newborn period. Seven subjects had other cardiac diagnoses.

### MRI protocol:

All participants (CHD and Control) underwent a fetal brain MRI during the third trimester using the same imaging protocol on a 3 Tesla MRI system equipped with a 32-channel cardiac coil (GE Medical Systems, Waukesha, WI). Sequences included a routine clinical single shot fast spin echo (SSFSE) T2 imaging. SSFSE parameters included TE = 100ms, TR = 4s, slice thickness = 3mm, matrix 256×192 with a field of view of 28–32 cm. SSFSE T2 imaging in multiple planes were used to create a 3D volume using slice-to-volume reconstruction which was then used to derive fetal total brain volume (TBV) using an automated pipeline [29–31].

After birth, only neonates with CHD underwent pre-operative brain MRI without sedation as soon as they could be safely transported to MRI as determined by the clinical team, typically within the first week of life. MRIs were again performed on the same scanner and included: 3D isotropic 1mm T1-weighted IR-SPGR (TE/TR 3.5/8.7ms, TI 450ms) and 3D 1mm isotropic T2 (TE/TR 93/3000 ms) [32]. The TBV on the neonatal pre-operative brain MRI was calculated from the T1 and T2 weighted images using the publicly available processing pipeline from the developing human connectome project [33, 34]. The neonatal MRIs were reviewed for the presence and severity of white matter injury (WMI) and classified as mild, moderate, or severe by a neuroradiologist blinded to all clinical outcomes as previously described [3]. Given our prior work demonstrating the clinical significance of moderate to severe WMI, this outcome was categorized as either normal/mild WMI vs. moderate-severe WMI [13].

### Primary predictors and outcomes:

Pregnant participants (CHD and control) completed home environment surveys at the time of enrollment, which collected information on self-reported race and ethnicity, household income, insurance type, parental education level, use of welfare or food stamps, maternal smoking history, household smoking exposure, and household exposure to recreational drug use. For income, participants were divided into household income levels above or below $75,000, which is below the low income level in the greater bay area [35]. A composite measure of an ‘at risk fetus’ was created if there was exposure to any of the following based on responses to the home environment survey: maternal smoking during pregnancy (defined as smoking occurring any time after the last menstrual period of the pregnancy), household exposure to either smoking or recreational drug use, use of welfare or food stamps, or low income as defined above. Community SDOH metrics were determined through the Child Opportunity Index (COI), which is comprised of 44 indicators in the domains of education, health and environment, and social and economic based on patient home address which was collected on the survey completed by pregnant participants. COI gives a 5-level scoring scale of very low through very high, which we collapsed into two categories: high (including high and very high), low (including low, very low, and moderate).

Our primary outcome was 1) Overall TBV across the fetal and neonatal MRIs adjusted for GA and fetal sex and 2) rate of change in TBV (i.e. slope) from fetal to neonatal MRI as a reflection of fetal brain growth. The secondary outcome was the presence of moderate to severe WMI after birth on the neonatal pre-operative brain MRI.

### Statistical analysis:

For our primary outcome of TBV across two time points and rate of change of TBV, repeated measures analysis utilizing generalized estimating equations was performed for each predictor variable in a univariable analysis for the CHD cohort. A multivariable repeated measures analysis was then performed based on the findings from the univariable analysis including variables with a p-value of < 0.1 and/or biologically plausible variables. The final model included fetal sex and gestational age at MRI as covariates. Analyses on fetal TBV as the primary outcome was performed separately for the control group to assess whether similar associations were identified in the control group. Control participants did not undergo postnatal MRI, thus repeated measures analyses were not performed for this group. Univariable and multivariable logistic regression was performed for the secondary outcome of the presence of moderate-severe WMI at birth for the CHD cohort. All statistical analyses were performed using STATA 16.0 software (StataCorp, LP, College Station, Texas, USA).

## Results

57 participants with fetal CHD were enrolled with a slight male predominance (n = 38, 66.7%). The majority had either TGA (n = 18, 31.6%) or SVP (n = 30, 52.6%). Seven (12.3%) had ‘other’ diagnoses consisting of coarctation, Tetralogy of Fallot or double outlet right ventricle. 24 control participants were enrolled. Baseline demographics and survey responses are listed in Table 1 for the entire cohort (CHD and control). There was a moderate non-response rate to certain survey questions. Among enrolled participants, 54 CHD participants (94.7%) and 24 control participants (100%) completed a fetal MRI at a mean gestational age of 33.9 weeks (95% CI: 33.7, 34.1) and 34.1 weeks (95% CI: 33.7, 34.5), respectively. One CHD participant could not complete the fetal MRI due to claustrophobia. After birth, 47 neonates in the CHD group (82.4%) completed a neonatal MRI at a mean gestational age of 39.3 weeks (95% CI: 38.9, 39.6). 10 did not complete a neonatal MRI due to clinical instability or scheduling issues prior to cardiac surgery. Morphometry to extract TBV could not be performed in 23 fetal MRIs and in three neonatal MRIs due to motion degradation leaving 33 (58.9%) fetal MRIs and 44 (93.6%) neonatal MRIs with TBV data for the analysis. Baseline demographics were not significantly different comparing those with successful vs. unsuccessful morphometry to extract TBV (supplemental tables 1 and 2).

For the primary outcome of TBV on the fetal and neonatal MRI, a univariable repeated measures analysis was performed for all variables. Table 2 includes results of overall TBV (across both the fetal and neonatal time points) as well as the rate of change in TBV for each week of GA (i.e. slope). Notably, several factors were associated with overall TBV and rate of growth including fetal sex, maternal smoking during pregnancy and/or exposure to household smoking or recreational drug use, use of welfare and/or food stamps, and poverty. The composite predictor variable of being an ‘at risk’ fetus was associated with a smaller overall TBV. The TBV was on average 13.9 mL smaller in at risk fetuses compared to those without risk factors (coeff: −13.9 mL, 95%CI: −28.8,0.9, p = 0.06). Similarly, the rate (i.e. slope) of change in TBV per week of gestational age was 3.l mL smaller among at risk fetuses compared to those without risk (coeff: −3.1 mL/week, 95%CI: −5.3,−0.9, p = 0.005) compared to fetuses without risk factors. Cardiac lesion, insurance status, maternal educational level, race/ethnicity, and COI were not associated with overall TBV or rate of change in TBV. No associations were noted between predictors and fetal TBV in the control group (supplemental Table 3)

**Figure 1.**
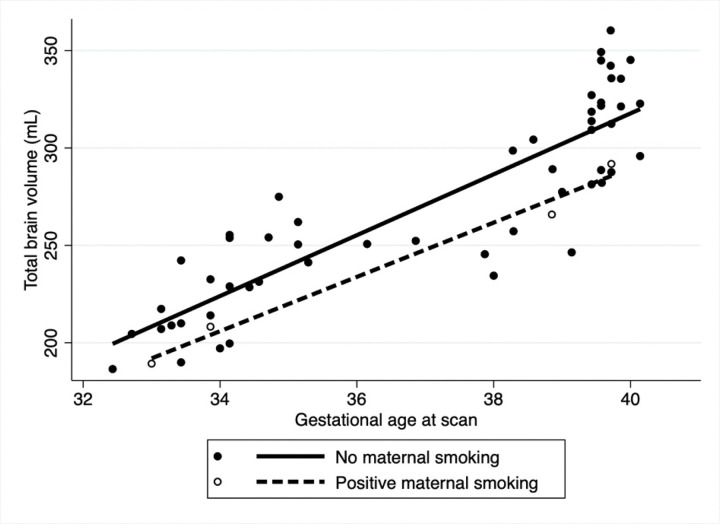
Scatter plot of total brain volume by gestational age at scan including both fetal and neonatal time points. In the multivariable analysis, after adjusting for fetal sex and gestational age at scan, fetuses whose mothers reported a personal history of smoking during pregnancy [MP1] (dashed line) had an overall lower TBV (coeff: −18.6, 95%CI: −29.9, −7.3, p= 0.001) and a slower rate of TBV growth per week of gestational age compared to those who denied smoking (solid line) (Coeff= −1.6 mL/week, 95%CI: −3.2, −0.05, p= 0.05) [MP1]Adjust the figure labels to distinguish between personal history of smoking (Fig 1) and household smoking (Fig 2)

**Figure 2.**
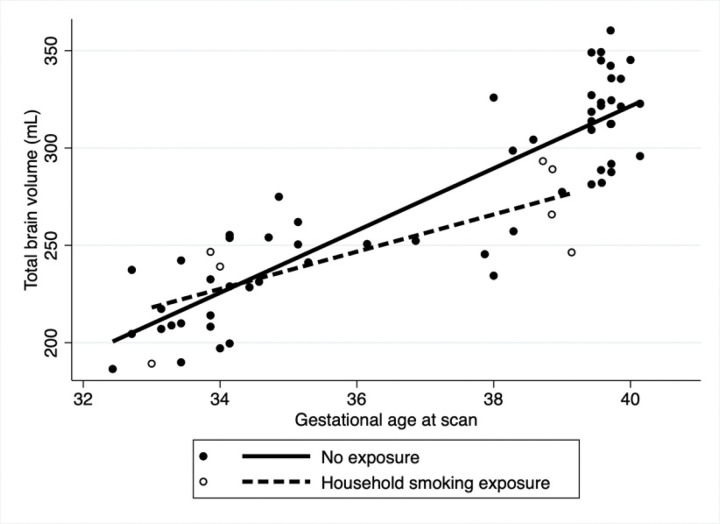
Scatter plot of total brain volume by gestational age at scan including both fetal and neonatal time points. In the multivariable analysis, after adjusting for fetal sex and gestational age at scan fetuses whose mothers reported exposure to household smoking or recreational drug use during pregnancy (dashed line) had a significantly slower rate of TBV growth per week of gestational age compared to those without exposure (solid line) (Coeff= −6.9 mL/week, 95%CI: −13.3, −0.4, p= 0.03). Overall TBV adjusted for week of GA was not significantly different across the groups (−9.9, 95%CI: −31.8, 12.1, p= 0.3).

**Figure 3.**
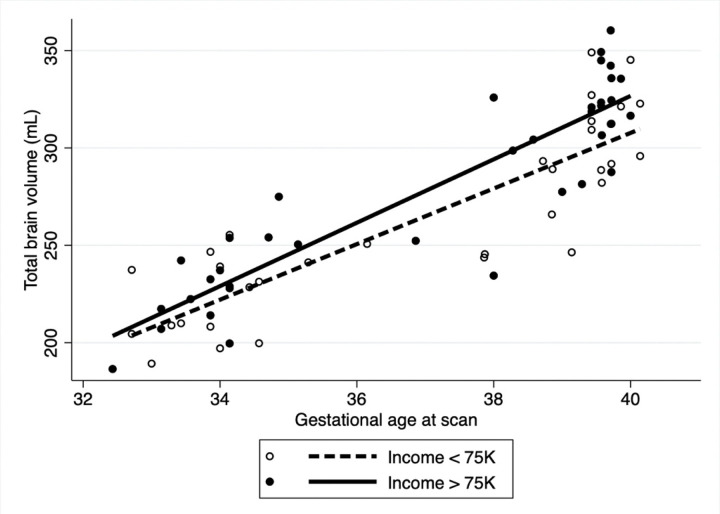
Scatter plot of total brain volume by gestational age at scan including both fetal and neonatal time points. After adjusting for fetal sex and gestational age at scan, fetuses whose mothers reported an income of > 75K (solid line) had a non-significant trend towards higher overall TBV (coeff: 11.4, 95%CI: −0.5, 23.4, p= 0.06) and a non-significant trend towards slower rate of TBV growth per week of gestational age compared to those with an income of < 75K (dashed line) (Coeff= 2.0 mL/week, 95%CI: −0.3, 4.4, p= 0.09).

**Figure 4.**
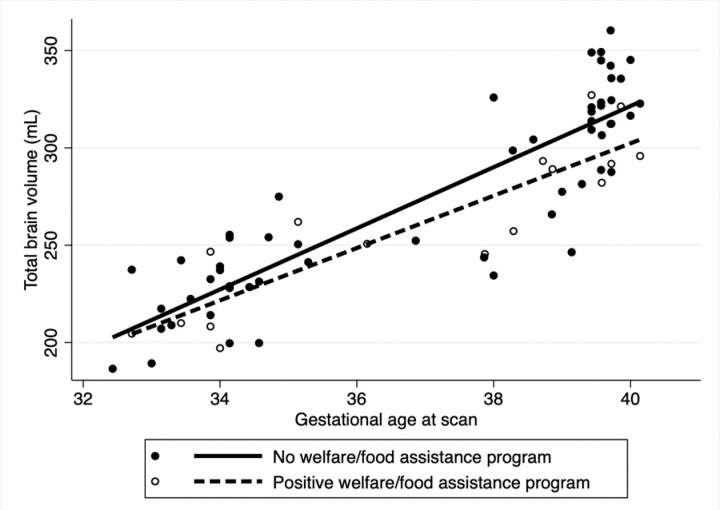
Scatter plot of total brain volume by gestational age at scan including both fetal and neonatal time points. After adjusting for fetal sex and gestational age at scan fetuses whose mothers reported use of welfare and/or a food assistance program during the pregnancy (dashed line) had a non-significant trend towards an overall lower TBV (−11.3, 95% CI: −23.6, −0.9, p= 0.06) and a non-significant trend towards slower rate of TBV growth per week of gestational age compared to those without welfare/food assistance program (solid line) (Coeff= −2.5 mL/week, 95%CI: −5.8, 0.7, p= 0.1).

**Figure 5.**
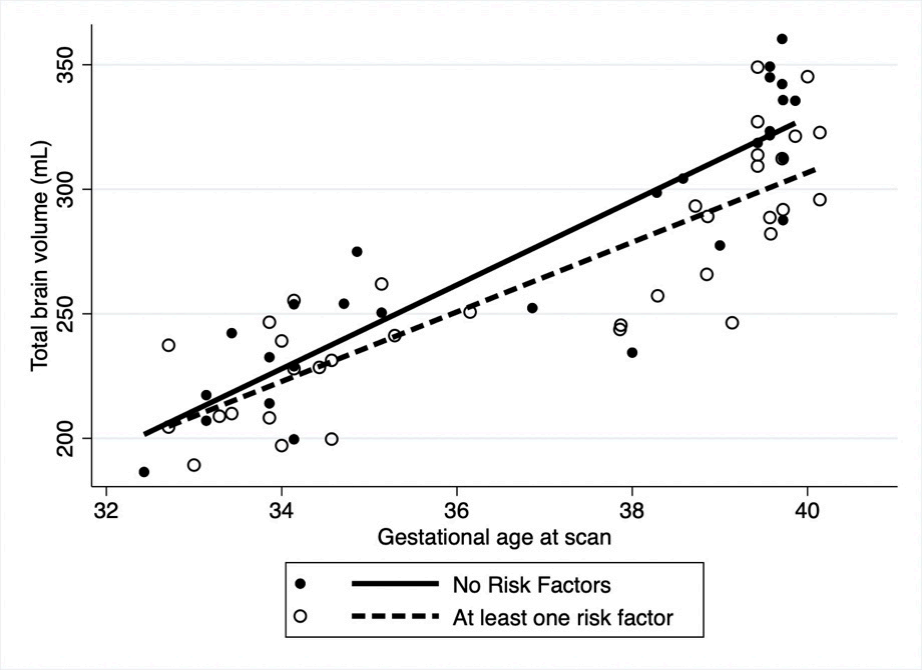
Scatter plot of total brain volume by gestational age at scan including both fetal and neonatal time points. After adjusting for fetal sex and gestational age at scan ‘At risk’ fetuses (dashed line) had much smaller overall TBV (coeff: −13.3, 95%CI: −25.5,−1.1 p= 0.03) and slower rate of brain growth (coeff: −2.5 mL/week, 95%CI: −5.0, −0.07, p= 0.04) compared to those without any risk factors.

After adjusting for fetal sex and GA at MRI in the multivariable analysis (Table 3), overall TBV was significantly lower for those who reported maternal smoking during pregnancy (coeff: −18.6, 95% CI: −29.9.3,−7.3 p = 0.001) with slower rate of brain growth (coeff: −1.6 95%CI: −3.2, 0.05, p = 0.05) compared to those that did not smoke (Fig. 1). Household exposure to smoking resulted in a slower rate of brain growth (coeff: −6.9, 95%CI: −13.3,−0.4 p = 0.03) compared to no exposure (Fig. 2). Similar trends were noted for income and use of welfare/food stamps though these did not achieve statistical significance (Figs. 3 and Fig. 4). Finally, fetuses ‘at risk’ had a much smaller overall TBV (coeff: −13.3, 95%CI: −25.5,−1.1 p = 0.03) and slower rate of brain growth (coeff: −2.5, 95%CI: −5.0, −0.07, p = 0.04) compared to those without any risk factors in the multivariable analysis (Fig. 5). Total brain volume was on average 13 mL smaller among at-risk fetuses compared to those without risk. For each week of gestational age, total brain volume grew at a rate 2.5 mL slower in the at-risk group compared to the group without any risk factors.

Rates of preoperative moderate to severe WMI by demographic and fetal home environmental factors are shown in Table 4. The frequency of moderate to severe WMI was significantly higher in neonates from low COI neighborhoods compared to high COI neighborhoods. After adjusting for gestational age at the time of neonatal MRI, the odds of moderate to severe pre-operative WMI was significantly lower in the patients from high COI neighborhoods compared to low COI (OR = 0.16, 95%CI: 0.03, 0.9, p = 0.04). Other predictors were not associated with risk of moderate to severe WMI.

## Discussion

This cohort study is one of the first investigating the impact of fetal environmental exposures and SDOH on fetal brain growth in patients with severe CHD. Our results demonstrate a significant effect of these factors on brain growth with both smaller overall brain volumes, slower rate of growth during this period and an increased risk of acquired postnatal WMI. Our results mirror prior studies on early life adversity and social disadvantage on neonatal brain volumes in other patient populations [36]. In addition to an underlying substrate of severe CHD, our study demonstrates potential environmental risks in the prenatal period that further contribute to ongoing brain growth and development.

The brain goes through a rapid phase of growth and development in the third trimester of fetal life and in the early neonatal period [37, 38], making this time particularly vulnerable to adverse exposures including abnormal cardiovascular physiology. It is well known that fetuses and neonates with severe CHD have less developed brains compared to those without CHD [4, 8, 39, 40], and several risk factors have been identified to explain this difference [14, 17, 27, 40]. In a previous study from our group, we demonstrated an ~ 24 mL difference in brain volume among third trimester fetuses with complex CHD compared to controls [27]. In this current study, overall TBV was ~ 13 mL smaller among CHD fetuses with at risk exposures compared to CHD fetuses without these exposures. Thus, although the magnitude of difference is not as great as having a substrate of CHD, our findings identify a potential link between several modifiable environmental factors such as smoking exposure, nutrition, and poverty and abnormal fetal brain growth. This provides important preliminary data that modifying exposures to these factors in utero may provide an opportunity for incremental improvement in brain growth in utero, increasing resilience towards additive risk factors that take place after birth.

We hypothesize that these environmental factors influence the developing fetus through placental changes among other pathways. Prior studies have shown numerous placental changes related to smoking exposure, including impaired placental development related to decreased vascularization, decreased vasculosyncytial membrane and cytotrophoblastic proliferation, and premature aging in smokers’ placentas, all of which may contribute to placental insufficiency and decreased nutrient and oxygen delivery to the fetus [41–43]. While smoking rates have decreased in recent years, a 2006 study demonstrated that 22% of reproductive age females are current smokers [44], making this an important fetal exposure for public health efforts. Other recreational drug exposures alter fetal and placental development through diverse mechanisms, many of which alter fetal brain development [45–47]. In general, placental abnormalities are common among fetuses with severe CHD with a wide array of observations including vascular abnormalities and inflammation [48, 49]. A limitation of our study is that it did not involve gross or histologic examination of the placenta to assess for these.

The use of welfare/food stamps appeared to have some relationship with fetal brain growth, but this was not statistically significant. It is possible that the use of food assistance programs may reflect nutritional status of the mother and fetus though we did not measure this specifically. In other populations, maternal nutrition, including both maternal obesity and malnutrition as well as specific nutrient deficiencies, have been linked with poor fetal growth and ND outcomes [50–53]. Thus, we plan to study this area in more detail in future studies with objective data on nutritional status during pregnancy in the CHD population as another potential target for intervention.

Interestingly, community metrics of SDOH as measured by the COI were not associated with fetal brain growth but lower COI was associated with a higher risk of pre-operative moderate to severe WMI after birth. As the COI is based on census tract data and patient home addresses, it is possible that some environmental factors as opposed to individual risk factors (i.e. smoking) may play a role in overall brain health. Certain environmental pollutants have been shown to affect regional brain growth, and prenatal particular air pollution exposure has been associated with worse neurodevelopmental outcomes, though a link between these factors and preoperative brain injury but not brain volume or growth seems unusual [53]. Further investigations in a larger sample size are needed to tease out the complexity of individual vs. environmental factors on the developing fetal brain and acquired brain injury.

### Limitations:

Although our study enrolled control participants, the sample was biased and reflected a majority White, high socioeconomic and high education population. The control participants only had a fetal brain MRI and no associations were found between the predictors and fetal TBV, likely secondary to the biased sample. Thus, we did not conduct additional analyses to evaluate for additive effects of environmental/socioeconomic factors by including group (CHD vs. control) as an interaction term. Our study is also limited by the relatively small sample size though participants had two imaging time points increasing our power for this study. There was a modest non-response rate on the survey which may bias our findings. Finally, it is important to note that our study design and analysis has demonstrated several interesting associations but does not establish causality.

## Conclusion

Exposure to smoking and some individual level social determinants of health influence fetal brain growth in severe CHD. Our findings identify candidate variables that confer potential risk in early life on brain health and neurodevelopmental outcomes in the CHD population. These findings will require replication in larger, diverse samples including a representative control population. Although the substrate of CHD still remains in this patient population, minimizing exposure to these variables may positively shift brain growth and development early in life allowing for some incremental improvements in neurodevelopmental outcomes and can be studied in future neuroprotective clinical trials.

## Supplementary Material

Supplementary Files

This is a list of supplementary files associated with this preprint. Click to download.

• SupplementalMaterial.docx

## Figures and Tables

**Table 1:** Baseline demographics of the study cohort and survey responses.

	Enrolled participants CHD (n = 57)	Enrolled participants Control (N = 24)
Male sex	38 (66.7%)	12 (50%)
Maternal race/eth		
NH White	16 (28.1%)	12 (50%)
NH Black	4 (7%)	0
Hispanic	24 (42.1%)	6 (25%)
Asian	7 (12.3%)	4 (16.7%)
Other	5 (8.8%)	2 (8.3%)
Cardiac Lesion		N/A
TGA	18 (31.6%)	
SVP	30 (52.6%)	
Other	7 (12.3%)	
Maternal insurance		
Public	15 (26.3%)	2 (8.7%)
Private	42 (73.7%)	21 (91.3%)
Maternal smoking history		
Yes	3 (5.3%)^a^	0
Household smoking/drug		
Yes	5 (8.8%)^b^	1/18 (5.6%)^e^
Welfare/food stamps		
Yes	12 (21.1%)^c^	1/22 (4.5%)^f^
Household Income		
< 75K	22 (38.6%)^d^	3/22 (13.6%)^g^
COI based on address		
Low	30 (52.6%)	3/23 (13.0%)
High	27 (47.4%)	20/23 (87.0%)
Maternal Education > HS		21/22 (95.4%)^h^

a: Missing information for 13 participants

b: Missing information for 7 participants

c: Missing information for 1 participant

d: Missing information for 5 participants

e: Missing information for 6 controls

f: Missing information for 2 controls

g: Missing information in 2 controls

h: Missing information in 2 controls

NH = non-Hispanic

TGA = Transposition of the Great Arteries

SVP = single ventricle physiology

**Table 2:** Repeated measures univariable analysis taking GA scan into account:

	Overall TBV (fetal and neonatal time points)	p-value	Rate of change in TBV (slope, mL/week)	p-value
Fetal sex male	23.5 (12.1,34.9)	< 0.001	0.1 (−2.4, 2.7)	0.9
Maternal race/eth		
NH White	Ref			
NH Black	−13.2 (−30.2,3.7)	0.13	2.6 (−3.2,8.4)	0.38
Hispanic	6.7 (−10.0,23.3)	0.43	−0.01 (−4.4,4.4)	1.0
Asian	−6.5 (−30.0,17.9)	0.59	1.7 (−2.7,6.1)	0.45
Other	−23.3 (−48.5,1.9)	0.07	−2.5 (−9.6,4.5)	0.48
Cardiac Lesion		
TGA	Ref		Ref	
SVP	−5.0 (−19.6,9.5)	0.5	0.08 (−2.3, 2.5)	0.9
Other	−4.5 (−26.3,17.2)	0.6	2.5 (−0.6,5.6)	0.2
Maternal insurance		
Public	Ref			
Private	5.7 (−9.3,20.8)	0.4	1.6 (−0.5,3.7)	0.2
Positive Maternal smoking history	−22.0 (−32.2,-11.8)	< 0.001	−1.6 (−3.3, -0.01)	0.05
Positive Household smoking/drug Exp	−15.1 (−37.6,7.4)	0.1	−6.4 (−11.6, -1.2)	0.02
Positive Welfare/food stamps	−12.8 (−25.5, -0.01)	0.05	−2.7 (−5.5, 0.08)	0.06
Household Income		
< 75K	Ref			
>75K	13.5 (−0.03, 27.0)	0.05	2.3 (0.20, 4.4)	0.03
Mat Education > HS	8.7 (−4.4, 21.8)	0.19	0.46 (−1.8,2.7)	0.7
COI		
Low	Ref			
High	8.6 (−4.6, 21.9)	0.2	0.8 (−1.3, 3.0)	0.4
At risk fetus	−13.9 (−28.8, 0.9)	0.06	−3.1 (−5.3, -0.9)	0.005

NH = non-Hispanic

TGA = Transposition of the Great Arteries

SVP = single ventricle physiology

COI= childhood opportunity index

**Table 3:** Repeated measures multivariable analysis for significant variables in Table 3 adjusted for GA scan and sex

	Overall TBV (fetal and neonatal time points)	p-value	Rate of change in TBV (slope, mL/week)	p-value
Positive Maternal smoking history	−18.6 (−29.9,-7.3)	0.001	−1.6 (−3.2, 0.05)	0.05
Positive Household smoking/drug Exp	−9.9 (−31.8, 12.1)	0.3	−6.9 (−13.3, −0.4)	0.03
Positive Welfare/food stamps	−11.3 (−23.6, −0.9)	0.06	−2.5 (−5.8, 0.7)	0.1
Household Income		
< 75K	Ref			
>75K	11.4 (−0.5, 23.4)	0.06	2.0 (−0.3, 4.4)	0.09
At risk fetus^a^	−13.3 (−25.5, −1.1)	0.03	−2.5 (−5.0, −0.07)	0.04

a: at risk fetus is if there was exposure to any of the following: maternal smoking during pregnancy, household exposure to smoking or drug use, use of welfare/food stamps, or poverty.

**Table 4:** Baseline demographics by presence of moderate-severe WMI

	None/Mild WMI N = 34	Mod-Sev WMI N = 11	p-value^a^
Fetal TBV, mL	224.3 (213.4, 235.2)	219.8 (196.7, 243.0)	0.7
Sex, Male	24 (70.6%)	8 (72.7%)	0.89
Cardiac Lesion			0.25
TGA	14 (41.2%)	2 (18.2%)	
SVP	15 (44.1%)	8 (72.7%)	
Other	5 (14.7%)	1 (9.1%)	
Maternal race/eth			0.24
NH White	11 (32.3%)	3 (27.3%)	
NH Black	2 (5.6%)	1 (9.1%)	
Hispanic	11 (32.3%)	7 (63.6%)	
Asian	6 (17.6%)	0	
Other	4 (11.8%)	0	
Maternal insurance, public	5/31 (16.1%)	4/9 (44.4%)	0.07
Positive Maternal smoking history	1/25 (4.0%)	1/10 (10.0%)	0.49
Positive Household smoking/drug Exp	3/29 (10.3%)	1/10 (10%)	0.97
Positive Welfare/food stamps	7/33 (21.2%)	3/11 (27.3%)	0.67
Household Income, < 75K	11/31 (35.5%)	7/11 (63.6%)	0.1
COI, low	14/34 (41.2%)	9/11 (81.8%)	0.02
Composite smoking	3/26 (11.5%)	2/10 (20%)	0.51
At risk fetus	12/26 (46.1%)	7/11 (63.6%)	0.33
GA birth, weeks	38.7 (38.4, 39.0)	38.5 (37.9, 39.1)	0.48
Birth Weight, Kg	3.3 (3.1,3.4)	3.3 (3.0, 3.5)	0.80
Neonatal TBV, mL	308.7 (295.7, 321.7)	292.2 (265.2, 319.2)	0.22

a: chi-squared test was used for categorical variables and two sample t-test was used for continuous variables.

TBV = total brain volume

COI = Childhood Opportunity Index

NH = non-Hispanic

TGA = Transposition of the Great Arteries

SVP = single ventricle physiology

## Data Availability

The data that support the findings of this study are not openly available due to the presence of PHI. De-identified data are available from the corresponding author upon reasonable request. Data are located in controlled access data storage in RedCap associated with the University of California San Francisco.
